# The Japanese version of the Phenomenological Control Scale

**DOI:** 10.1093/nc/niaf008

**Published:** 2025-05-21

**Authors:** Shu Imaizumi, Keisuke Suzuki

**Affiliations:** Institute for Education and Human Development, Ochanomizu University, 2-1-1 Otsuka, Bunkyo, Tokyo 112-8610, Japan; Center for Human Nature, Artificial Intelligence, and Neuroscience, Hokkaido University, Kita 12, Nishi 7, Kita-ku, Sapporo, Hokkaido 060-0812, Japan

**Keywords:** phenomenological control, hypnosis, perception, imagery, inter-individual variations, scale development

## Abstract

People vary in their capacity for phenomenological control, which enables them to align their perceptual experiences with their intentions and goals. The Phenomenological Control Scale was developed to measure this trait, and we developed and validated a Japanese version of this scale (PCS-J) based on preregistered online surveys (*n* = 261; retest *n* = 152). The PCS-J demonstrated sufficient internal consistency and test–retest reliability. Given the known association between hypnotic susceptibility and positive schizotypy, the convergent validity of the PCS-J was supported by a weak positive correlation with positive schizotypy. The discriminant validity of the PCS-J was demonstrated by the absence of a correlation with negative schizotypy. The PCS-J would be useful for research on perception, phenomenological control, and their individual differences in Japanese samples, as well as for intercultural studies.

## Introduction

The human mind is an intricate construct capable of altering perceptions and cognitive processes in various ways. This intrinsic flexibility underlying the various states of consciousness is not only found in mental disorders, such as psychosis where the connection with reality is distorted, but also in healthy individuals. This aspect of the human mind has been extensively documented in historical and clinical research, including research into hypnosis, a phenomenon showcasing the spectrum of consciousness and malleability of how people experience reality (Kihlstrom 1985, Pintar and Lynn 2008).

Hypnotic suggestion is preceded by hypnotic induction, which primes individuals to become more receptive to suggestions (Green et al. 2005, Terhune and Cardena 2016). Verbal suggestions given during hypnosis can influence a broad spectrum of perceptual and cognitive experiences, such as the alleviation of pain (Milling et al. 2021), alterations in inhibitory control (Raz et al. 2006), amnesia (Kihlstrom 1997), and diminished agency (Lush et al. 2017, Polito et al. 2018). These effects are characterized by involuntariness. While experiences stemming from hypnotic suggestions are self-generated, individuals undergoing them may not be aware that they are producing these effects.

The degree of receptiveness to hypnotic suggestion (i.e. hypnotic susceptibility) varies among individuals but remains stable within an individual over their lifetime (Piccione et al. 1989). Hypnotic susceptibility has no or a weak association with personality traits (Dermen and London 1965, Zhang et al. 2017), cognitive inhibition, and dissociative tendencies (Dienes et al. 2009). While there is some evidence linking it to schizotypy and delusional proneness (Connors et al. 2014), associations between hypnotic susceptibility and other traits may be reduced or eliminated when controlling for demand characteristics and contextual effects (Lynn et al. 2020). Notably, fantasy proneness remains associated with hypnotic susceptibility even when such controls are applied (Silva and Kirsch 1992).

Hypnotic suggestion can produce effects that do not necessarily require a trance state (Barber and Glass 1962, Meyer and Lynn 2011). Researchers are now attempting to conceptualize hypnotic susceptibility as a form of phenomenological control—essentially, the ability to generate perceptual and cognitive experiences in line with intentions and goals (Lush et al. 2021, Dienes et al. 2022). Clarifying the concept and mechanisms of phenomenological control is pertinent to discussions on cognitive penetration (Oakley and Halligan 2013) and may provide practical evidence for predictive coding that accounts for perception (Martin and Pacherie 2019; but see Lush et al. 2023 for a critique of the inadequacy of predictive processing accounts of phenomenological control). Furthermore, studying the voluntary perceptual and cognitive changes that occur without explicit linguistic cues and the role of unconscious processes is important for exploring the interaction between individual cognition and social context. This approach is also crucial for mitigating the influence of demand characteristics (Orne 1962) in experiments. Demand characteristics may generate experiences based on participants’ perceptions of what the experimenter expects, which may be inconsistent with the intent of the experiment.

As with hypnotic susceptibility, the capacity for phenomenological control varies among individuals. The Phenomenological Control Scale (PCS), a single-factor 10-item scale, was developed to measure this capacity (Lush et al. 2021). The PCS is adapted from the Sussex–Waterloo Scale of Hypnotizability (SWASH) (Lush et al. 2018), which is an adaptation of the Waterloo-Stanford Group Scale of Hypnotic Suggestibility, Form C (Bowers 1993). The latter is a group adaptation of the Stanford Hypnotic Susceptibility Scale, Form C (Weitzenhoffer and Hilgard 1962). In administering the PCS, participants are first given spoken suggestions that prompt them to imaginatively change perceptual, cognitive, and motor controls, but not in the context of hypnosis. Then, participants’ changes in perceptual and cognitive experiences and motor control are measured subjectively or objectively. While the reliability of PCS subjective scores has been confirmed, the reliability of the objective scores is low and not recommended for use (Lush et al. 2021). Therefore, the present study focused only on PCS subjective scores.

The purpose of the present study was to develop a Japanese version of the PCS (PCS-J) and test its reliability and validity to enable future research on phenomenological control in Japan and cross-cultural comparisons using the PCS. We also aimed to enable online studies using the PCS-J, as in a previous study (Palfi et al. 2020). To achieve online implementation, the suggestive instructions were provided using recorded speech. Two online surveys were conducted to validate the PCS-J.

## Methods and materials

### Scale translation

We obtained permission from the original author to translate the PCS into Japanese. The second author translated the original version into Japanese as a draft. The first author then reviewed and revised the draft. This tentative Japanese version was approved by both authors and back-translated into English by professional translators who were blinded to our purpose and the original version. The original author reviewed the consistency between the original and back-translated versions and recommended adding minor revisions to the wording. The authors made minor revisions to the Japanese and back-translated versions. The back-translators approved the consistency between both versions following these minor revisions. The original author approved the revised back-translated version. Three psychology students who were native Japanese speakers completed and reviewed the revised tentative Japanese version. Some of them noted typographical errors in the revised tentative Japanese version, but they all approved the comprehensibility of this version’s instructions and response scale. After amending the scale for typographical errors, we employed this as the final version of the PCS-J.

### Design of validation study

The study design was preregistered (https://osf.io/3cakh) and approved by the ethics committee of the Center for Experimental Research in Social Sciences, Hokkaido University (FY2022-12). The reliability of the PCS-J was examined in terms of internal consistency and test–retest reliability. Hypnotic susceptibility, a construct relevant to phenomenological control (Lush et al. 2021), weakly and positively correlates with positive schizotypal personalities, but less so with negative and disorganized schizotypal personalities (Jamieson and Gruzelier 2001, Connors et al. 2014). Schizotypal personality refers to psychological traits that resemble behavioral, cognitive, and emotional disturbances characteristic of schizophrenia but manifest at a subclinical level (Ettinger et al. 2015). We expected that phenomenological control would also show similar (null) correlations with schizotypal personalities. Therefore, the construct validity of the PCS-J was examined by correlations with positive, negative, and disorganized schizotypal personalities, as measured using the Japanese version of the Schizotypal Personality Questionnaire Brief (SPQ-B) (Raine and Benishay 1995, Ito et al. 2008). The convergent validity of the PCS-J was assumed to appear as a positive correlation between PCS-J and positive schizotypal personality scores. The discriminant validity of the PCS-J was assumed to appear in the correlations between the scores for the PCS-J and negative and disorganized schizotypal personality, which are weaker than the correlation with positive schizotypal personality. Finally, since the PCS-J is administered online, we tested if the representative value of the PCS-J was comparable with those of the original PCS in an in-person setting (Lush et al. 2021) and of the SWASH (Lush et al. 2018), which is substantially relevant to the PCS, in an online setting (Palfi et al. 2020).

### Participants

The targeted sample size in Survey 1 was 244, the same as in the PCS development study (Lush et al. 2021), and sufficient to detect a correlation effect size of 0.226 with a statistical power of 0.95 and a two-sided alpha of 0.05, according to a sensitivity analysis using G*Power 3.1.9.7 (Faul et al. 2007). Since Connors et al. (2014) reported a significant correlation between hypnotizability and positive schizotypal personality (*r* = 0.254), our sample size was sufficient for convergent validity analysis.

Native and fluent Japanese speakers were recruited through CrowdWorks (https://crowdworks.jp/), a crowdsourcing platform in Japan. Survey 1 was conducted on 21 November 2022, and Survey 2 on 21 December 2022. A total of 330 participants took part in Survey 1. The participants completed surveys using Gorilla Experiment Builder (Anwyl-Irvine et al. 2020) on their own computers while listening to instructions through their earphones or headphones. Each participant provided electronic informed consent before the survey. The participants were paid 330 Japanese yen for completing the survey.

Participants who did not pay attention to or follow the instructions were excluded from the analysis to eliminate unreliable responses and measurement error (Oppenheimer et al. 2009, Thomas and Clifford 2017). Specifically, we excluded participants who answered “no” to either of the following questions: “In this experiment, you were instructed to extend your arms out in front of you, palms facing each other. When you did so, did you actually move your body in that manner?” and “When you were instructed to close your eyes during the experiment, did you actually close your eyes?” We also excluded participants who failed to follow the instructions for the question, “Do you have any hobbies that you are passionate about? Choose both ‘Not so much’ and ‘Strongly agree’.” The first two questions were asked at the end of the PCS-J, while the last one was asked at the end of each survey. Additionally, participants who provided inconsistent responses for their gender and age between the two surveys were excluded due to the possibility of false responses. However, an age increase of one year was accepted.

If the number of valid responses in Survey 1 fell below 244 based on the above exclusion criteria, we planned to add samples until the number of valid responses exceeded 244. However, 69 participants were excluded from the initial 330 in Survey 1, making it unnecessary to increase the sample size. Data from 261 participants in Survey 1 (141 men, 117 women, and 3 unknown; mean age of 42.2 years, *SD* = 9.2, range 22–65) were included in the analyses. These 261 participants were invited to Survey 2, and 253 of them completed it. Of these, 101 participants were excluded based on the same criteria. Consequently, data from 152 participants (92 men and 60 women; mean age of 42.5 years, *SD* = 9.3, range 22–64) were analyzed to assess the test-retest reliability of the PCS-J.

### Measures

The PCS-J consists of a script for instructions and 10 items asking about subjective experiences during the instructions (Table 1). The full version of the script and items in Japanese can be found in the Supplementary Material and on the Open Science Framework (https://osf.io/74s69/). The instructions were provided using the artificial voice of a female Japanese speaker. Artificially generated speech was used to create controlled stimuli with clear and neutral intonation. After the instructions, each item was rated on a six-point Likert scale ranging from 0 to 5. The item and scale scores of the PCS-J were calculated according to the scoring methods of the Subjective Scale of the original PCS (Lush et al. 2021). Before calculating the scale score, the score for item 4 was calculated by averaging the responses to sub-items 4a (“sweet”) and 4b (“sour”). The score for item 10 was a geometric mean of the responses to sub-items 10a (“urge-to-press”) and 10b (“no-memory”). The PCS-J scale score is the mean of the scores of all 10 items. A higher score indicates a greater capacity for phenomenological control.

**Table 1.** Descriptive statistics of the PCS-J items (*n* = 261).

Item	Mean	*SD*	*α*
**1. Hand lowering**. How strongly did you feel your hand becoming heavy, where 0 means you felt your arm was no more heavy than normal and 5 means you felt your arm becoming as heavy as if you had a heavy object in your hand, pulling it down?	2.97	1.46	0.790 [0.754, 0.826]
**2. Moving hands together**. How strongly did you feel a force between your hands, where 0 means you felt no force at all and 5 means you felt a force so strong it was as if your hands were real magnets?	2.44	1.46	0.791 [0.756, 0.826]
**3. Mosquito hallucination**. How strongly did you feel the sensation of a mosquito being there, in either sound or touch, where 0 means you felt no sensation and 5 means you felt by any means as if there actually was a mosquito there?	1.77	1.46	0.800 [0.766, 0.833]
**4. Taste hallucination**. (a) How strongly did you taste a SWEET taste in your mouth, where 0 means you felt no taste at all and 5 means you felt a strong taste? (b) How strongly did you taste a SOUR taste in your mouth, where 0 means you felt no taste at all and 5 means you felt a strong taste?	2.15	1.45	0.795 [0.758, 0.831]
**5. Arm rigidity**. How stiff did your arm feel, where 0 means no more stiffness than normal and 5 means you could feel a stiffness so compelling no amount of effort would overcome it?	2.60	1.54	0.786 [0.749, 0.822]
**6. Arm immobilisation**. How strongly did you feel a heaviness in your hand, where 0 means you felt no heaviness at all and 5 means your hand felt so heavy it was as if a very heavy object was actually pressing it down?	2.39	1.45	0.784 [0.749, 0.820]
**7. Music hallucination**. Report how clearly you heard the music, where 0 means you did not hear any music at all and 5 means you heard it so clearly it was as though it was coming from the best sound system.	0.65	1.17	0.813 [0.783, 0.844]
**8. Negative visual hallucination**. How invisible was a third ball, where 0 means you saw three balls clearly, and 5 means you only saw two balls, and any number in between means you had some difficulty in seeing a third ball?	0.63	1.25	0.825 [0.797, 0.854]
**9. Amnesia**. How hard was it to remember events before you were told “now you can remember everything”, where 0 means you could remember events as easily as normal and 5 means you found it so difficult to remember it was as if there was an actual blank in your memory?	1.68	1.21	0.824 [0.795, 0.853]
**10. Post-session suggestion**. (a) Report how strong an urge you felt to press the space bar, where 0 means you had no urge whatsoever and 5 means you had a clear urge to press the space bar repeatedly. (b) Report how clearly you remembered being given the instruction to press the space bar six times, where 0 means you were able at that time to remember the instruction normally and 5 means you had no memory of the instruction at that time.	0.89	1.19	0.822 [0.794, 0.851]

All items are prefixed with “On a scale from 0 to 5…” The English version was adopted from Lush et al. (2021). The α indicates the if-item-dropped Cronbach’s alpha, and its 95% confidence intervals are in square brackets.

The SPQ-B scale and subscale scores were calculated as described by Ito et al. (2008). The “yes” and “no” responses to each item were scored as 1 and 0, respectively. The sum of the item scores served as the scale and subscale scores. The SPQ-B consists of three subscales: eight items on the Cognitive-Perceptual subscale corresponding to positive schizotypal personality, eight items on the Interpersonal subscale corresponding to negative schizotypal personality, and six items on the Disorganized subscale corresponding to disorganized schizotypal personality. Higher scores indicate greater schizotypy. Participants completed the SPQ-B only in Survey 1.

### Preregistered analysis

Descriptive statistics of the PCS-J and SPQ-B, including the mean, *SD*, minimum, maximum, kurtosis, and skewness, were analyzed. We also presented the PCS-J scale score distribution for Survey 1. The internal consistency of the PCS-J was assessed using Cronbach’s alpha with a 95% confidence interval (CI) and individual item reliability (i.e. if-item-dropped alpha). 95% CI can be interpreted as 95% credible interval (CrI) assuming a uniform prior. Cronbach’s alpha was also analyzed for the scale and subscale scores of the SPQ-B for descriptive purposes. The test–retest reliability of the PCS-J was assessed using a Bayesian paired-sample two-tailed *t*-test to examine the equivalence between the PCS-J scale scores in both surveys. Effect size of delta with 95% CrI was assessed using Cauchy prior distribution with a scale of 0.707. A Bayes factor for the alternative hypothesis (BF_10_) of smaller than 0.33 and 0.10 was interpreted as moderate and strong evidence of equivalence of both surveys, respectively (Jeffreys 1998). To further check the test-retest reliability, a Bayesian two-sided correlation analysis was performed for the PCS-J scale scores in both surveys. Pearson’s rho with a 95% CrI was assessed using a stretched beta prior width of 1. BF_10_ values greater than 3 and 10 were interpreted as moderate and strong evidence of correlation, respectively.

To assess the convergent validity of the PCS-J, a Bayesian two-sided correlation analysis was performed to determine whether the PCS-J scale score correlated with the SPQ-B scale score and the Cognitive-Perceptual subscale score. To assess discriminant validity, we also tested whether the PCS-J scale score correlated with the Interpersonal and Disorganized subscale scores. Pearson’s rho with a 95% CrI was assessed using a stretched beta prior width of 1. BF_10_ values greater than 3 and 10 were interpreted as moderate and strong evidence of correlation, respectively. BF_10_ of smaller than 0.33 and 0.10 was interpreted as moderate and strong evidence of a lack of correlation, respectively.

Finally, we tested whether the PCS-J results were comparable to the PCS subjective scores in an in-person session (Lush et al. 2021) and to the SWASH subjective scores in an online session (Palfi et al. 2020). Bayesian analysis of variance (ANOVA) with the study (our Survey 1, Lush et al. 2021 and Palfi et al. 2020) as a fixed factor was performed on the scale scores ranging from 0 to 5. The Bayes factor for a model including the study factor against the null model (BF_m_) with an *r* scale for the fixed effect of 0.5 was assessed. BF_m_ values smaller than 0.33 and 0.10 were interpreted as moderate and strong evidence of a null model (i.e. suggesting no difference between studies), respectively. The *R*^2^ with 95% CrI was reported as the effect size of the ANOVA. Post hoc tests were performed if we found moderate evidence in favor of a model that included the study factor (i.e. BF_m_ > 3). Evidence of (null) differences between studies was evaluated based on the BF_10_ for each pair according to the above criteria (i.e. larger than 3 or less than 0.33).

Statistical analyses were performed by using JASP 0.19.2 (JASP Team 2024). The above analysis used the default prior distributions and scale factors provided by JASP.

### Exploratory analysis

Robustness checks for BF_10_ for correlation analysis and *t*-test are reported as Figures S1–S9 in the Supplementary Material.

## Results and discussion

### Score distribution

**Figure 1:**
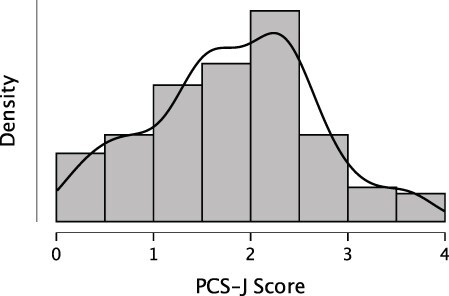
Distribution of the PCS-J scale score.

Tables 1 and 2 summarize item and scale scores of the PCS-J and (sub)scale scores of the SPQ-B. A histogram of the PCS-J scale scores from Survey 1 (*n* = 261) is shown in Fig. 1. Although the Shapiro–Wilk test suggested a non-normal distribution of PCS-J scores in Survey 1 (*p* = 0.016), the scores were distributed from low to high, with one peak in the center, according to the visual inspection of Fig. 1. The mean plus or minus 1 *SD* of the PCS-J scale score was within the range of 0 to 5, suggesting no floor or ceiling effects. These results suggest that the PCS-J is likely to reflect inter-individual variations.

**Table 2.** Descriptive statistics of the PCS-J and SPQ-B.

	Mean	*SD*	Range	Kurtosis	Skewness	*α* [95% CI]
PCS-J						
Survey 1 (*n* = 261)	1.82	0.85	0.00–3.85	−0.38	−0.03	0.820 [0.786, 0.850]
Survey 1 (*n* = 152)*	1.77	0.86	0.00–3.85	−0.45	0.06	0.827 [0.784, 0.863]
Survey 2 (*n* = 152)	1.73	0.89	0.10–4.00	−0.61	−0.02	0.849 [0.812, 0.880]
SPQ-B						
Total	9.44	4.43	0–21	−0.36	−0.06	0.817 [0.783, 0.847]
Cognitive-Perceptual	2.00	1.72	0–8	0.28	0.81	0.583 [0.500, 0.655]
Interpersonal	5.23	2.00	0–8	0.22	−0.81	0.686 [0.624, 0.740]
Disorganized	2.21	1.72	0–6	−0.88	0.38	0.678 [0.613, 0.734]

The asterisk indicates a subsample of the participants in Survey 1, who were included in the test–retest reliability analysis.

### Reliability

Cronbach’s alpha for the PCS-J was 0.820 in Survey 1 and 0.849 in Survey 2 (Table 2). The lower limits of their 95% CIs of 0.786 and 0.812, respectively, were higher than the upper limit of the 95% CI of the original PCS subjective score (alpha = 0.68, 95% CI [0.62, 0.74]) (Lush et al. 2021). These results suggest that the PCS-J has adequate internal consistency, and its Cronbach’s alpha was higher than that of the PCS.

**Figure 2:**
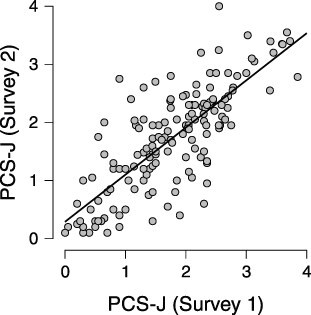
Test–retest correlation of the PCS-J scale score.

We found moderate evidence of equivalence in the PCS-J scale scores between both surveys (BF_10_ = 0.14, *δ* = 0.08, 95% CrI [−0.08, 0.23]; Figure S1). Moreover, as shown in Fig. 2, we found evidence of a strong positive correlation between both surveys (*r* = 0.781, 95% CrI [0.705, 0.834], BF_10_ = 1.66 × 10^29^; Figure S2). These results suggest the PCS-J has adequate test–retest reliability.

### Construct validity

**Figure 3:**
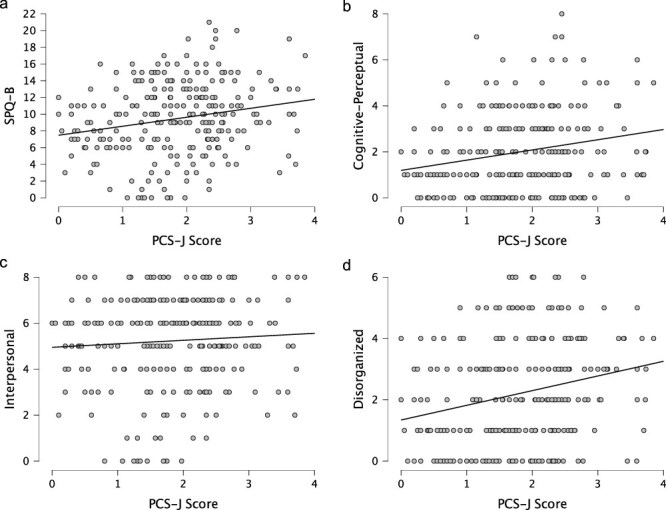
Scatterplots between the PCS-J, (a) SPQ-B, and (b) the Cognitive-Perceptual, (c) Interpersonal, and (d) Disorganized subscales.

The PCS-J scale score was weakly and positively correlated with the SPQ-B scale score (*r* = 0.206, 95% CrI [0.086, 0.317], BF_10_ = 19.92, Fig. 3a and S3) and the Cognitive-Perceptual subscale score (i.e. positive schizotypal personality; *r* = 0.219, 95% CrI [0.100, 0.330], BF_10_ = 43.66, Fig. 3b and S4) with strong evidence. These results are consistent with correlations previously observed between hypnotic susceptibility and positive schizotypal personality (Jamieson and Gruzelier 2001, Connors et al. 2014), suggesting the convergent validity of the PCS-J.

The PCS-J did not correlate with the Interpersonal subscale (i.e. negative schizotypal personality; *r* = 0.064, 95% CrI [–0.057, 0.183], BF_10_ = 0.13, Fig. 3c and S5) with moderate evidence. This result is consistent with previously reported null correlations between hypnotic susceptibility and negative schizotypal personality (Connors et al. 2014) and suggests the discriminant validity of the PCS-J.

Contrary to our prediction and a very weak correlation as previously reported (Connors et al. 2014), we found strong evidence of a positive correlation between the PCS-J scale score and the Disorganized subscale score (*r* = 0.236, 95% CrI [0.117, 0.345], BF_10_ = 119.66, Fig. 3d and S6).

### Comparison with English versions

**Figure 4:**
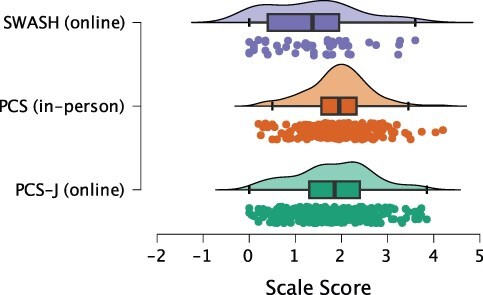
Comparisons between relevant scales. SWASH and PCS data retrieved from open materials of Palfi et al. (2020) and Lush et al. (2021), respectively.

We found strong evidence of differences between the PCS-J (*n* =261, see Table 2), original PCS in an in-person setting (*n* = 244, Lush et al. 2021), and SWASH online (*n *= 45, Palfi et al. 2020) (BF_m_ = 227.58, *R*^2^ = 0.033, 95% CrI [0.013, 0.059]; Fig. 4). Post hoc tests provided strong evidence of lower scores for the SWASH (mean = 1.36, *SD* = 0.99) than for the PCS-J (BF_10_ = 22.28, *δ* = 0.49, 95% CrI [0.18, 0.81]; Figure S7) and PCS (mean = 1.93, *SD* = 0.67, BF_10_ = 7066.39, *δ* = 0.75, 95% CrI [0.43, 1.08]; Figure S8) consistent with a previous study (Lush et al. 2021), and inconclusive evidence for the difference between the PCS-J and PCS (BF_10_ = 0.39, *δ* = −0.15, 95% CrI [−0.32, 0.03]; Figure S9).

We speculate that the presence or absence of a hypnotic context could explain the lower score for the SWASH online than for the PCS-J. These results are consistent with previous findings that responses are stronger when suggestions are presented as imaginative rather than hypnotic (Scacchia and De Pascalis 2020); however, it is worth noting that the opposite effect has also been reported (Braffman and Kirsch 1999, Gandhi and Oakley 2005). In the PCS development study (Lush et al. 2021), preconceived notions of hypnosis may have prompted participants to resist hypnotic induction during the SWASH measurement. Nevertheless, we should be cautious about sample differences (e.g. languages and sampling sites) as potential factors confounding the effects of scale and administration format.

## Conclusions

The present study developed a Japanese version of the PCS to measure traits of phenomenological control in healthy individuals in an online setting without a hypnotic context. The internal consistency and test–retest reliability of the PCS-J were adequate. The construct validity of the PCS-J was supported by the (null) correlations with schizotypal personality. Nevertheless, further validation of the PCS-J with scales measuring hypnotic susceptibility and fantasy proneness (e.g. Silva and Kirsch 1992), which are expected to be robustly related to phenomenological control capacity, would be beneficial. Additionally, validation in an in-person setting would provide further support. The PCS-J would be useful for research on human perception, phenomenological control, and inter-individual variations in Japanese samples, and for future intercultural studies. We also hope that this study will encourage the development of other non-English versions of the PCS.

## Supplementary Material

niaf008_Supp

## Data Availability

The raw data associated with this study, along with the instructional scripts, items, and instructional audio files for the PCS-J, can be found in the Supplementary Material and on the Open Science Framework (https://osf.io/74s69/). The program to conduct online administration of the PCS-J is available at GitHub (https://github.com/ksk-S/PhenoControl-JP).
