# Supplementary material for: The Japanese version of the Phenomenological Control Scale
Source: Neurosci Conscious. 2025 May 21;2025(1):niaf008. doi: 10.1093/nc/niaf008 (PMC12094076; doi:10.1093/nc/niaf008)
Supplement: niaf008_Supp [file niaf008_supp.zip › suppl_data/PCS-J_Questionnaire_v2.pdf]

The Japanese version of the Phenomenological Control Scale
日本語版現象学的制御尺度

画面に提示される教示文と質問紙

備考

- 次頁以降における斜体は注釈であり、画面に提示されない。
- 本尺度の終盤で尋ねられる以下の 2 問は、原版 (Lush et al., 2021) には含まれず、参加者が指示に従っていたかどうかを確認することを目的として日本語版に追加された。

  「本日の実験の中で、両腕を前に伸ばして手のひらを向かい合わせにするよう指示しました。その際に、実際にそのように体を動かしましたか？」

  「実験の中で目を閉じるように指示された際に、実際に目を閉じましたか？」

参考文献

Lush, P., & Dienes, Z. (2022, April 20). Phenomenological Control Scale norms. Retrieved from https://osf.io/4x25a/

Lush, P., Scott, R. B., Seth, A. K., & Dienes, Z. (2021). The Phenomenological Control Scale: Measuring the capacity for creating illusory nonvolition, hallucination and delusion. *Collabra: Psychology, 7*(1), 29542. https://doi.org/10.1525/collabra.29542

ヘッドホンかイヤホンをつけてから「次へ」を押して、実験の説明をお聞きください。

----------改ページ----------

まもなく音声による指示が再生されます。
音声を注意深く聞いて、指示に従ってください。
「次へ」を押すと音声が始まります。
（音声が始まるまで、少し時間がかかることがあります。）

----------改ページ----------
1.導入.wav と 2.暗示.wav と 3.幻視はじめに.wav を再生

音声が再生中です
（音声を注意深く聞いて、指示にしたがってください）

----------改ページ----------
4.幻視ボール.wav を再生し、カラーボール画像 (https://osf.io/sc4d2) を画面に提示

----------改ページ----------
5.幻視目を開ける.wav を再生

音声が再生中です
（音声を注意深く聞いて、指示にしたがってください）

----------改ページ----------
6.幻視入力.wav を再生

ボールの色を入力してください。
ボールの色を入力し終えたら、「完了」を押してください。

----------改ページ----------
7.1.記憶喪失 1.wav を再生

音声が再生中です
（音声を注意深く聞いて、指示にしたがってください）

----------改ページ----------

（お待ち下さい）

----------改ページ----------

この実験を始めてから今までに起こったことを簡単に書き出してください。細部まで書く必要はありません。２分間で書き終えてください。時間がきたら自動的に次に進みます。

----------改ページ----------

8.思い出せる.wav を再生

音声が再生中です

（音声を注意深く聞いて、指示にしたがってください）

----------改ページ----------

この実験を始めてから今までに起こったことを簡単に書き出してください。細部まで書く必要はありません。２分間で書き終えてください。時間がきたら自動的に次に進みます。

----------改ページ----------

これから、実験中に想像するように指示された具体的な出来事を、時系列にそってお見せしていきます。それぞれの想像するように暗示された経験について、主観的な評価、つまり、その暗示による効果をどの程度強く感じたかを記入していただきます。

場合によっては、暗示の効果を正確に評価するのが難しく、当てずっぽうになってしまうかもしれません。それでも、あなたが最も合っていると思うものをお選びください。

0 から 5 までの数字を入力することで、それぞれの出来事に対するあなたの主観的な評価を答えてもらいます。0 から 5 までの数字が何を意味するかは、それぞれの画面で説明されます。

----------改ページ----------

最初にあなたは、前に出した手が重くなって下がっていくと指示されました。0 から 5 までの尺度で、どの程度手が重くなったと感じましたか？

ここで、0 は腕の重さがいつもと全く変わらなく感じたことを、5 は実際に重いものを持っ

たのと同じように腕が重くなったと感じたことを意味します。

あなたの体験に対応する数字を選択してください

通常の重さだった 0, 1, 2, 3, 4, 5　非常に重かった

----------改ページ----------

次に、あなたは両手を 30cm ほど離して前に出し、両手を引き寄せ合う力を想像してくださいと指示されました。

0 から 5 までの尺度で、どのくらい強く力を感じましたか？0 は力を全く感じなかった場合、5 は手が磁石になったような力を感じた場合を表しています。

あなたの体験に対応する数字を選択してください

力を感じなかった　0, 1, 2, 3, 4, 5　強い力を感じた

----------改ページ----------

次に、蚊の羽音に気づくかと言われ、蚊が煩わしかったら払いのけるように言われました。

0 から 5 までの尺度で、音や触感として、どのくらい強く蚊がそこにいる感覚がありましたか？0 は全くそう感じなかった、5 はまるで本当に蚊がいるとしか思えないような感覚だったことを表します。

あなたの体験に対応する数字を選択してください

蚊を全く感じなかった　0, 1, 2, 3, 4, 5　本物の蚊のような感覚があった

----------改ページ----------

次に、あなたは口の中で「甘い味」がすると言われました。

0 から 5 までの尺度で、どのくらい強く甘い味を感じましたか？0 は全く感じなかった、5 は強い甘みを感じたことを表しています。

あなたの体験に対応する数字を選択してください

全く味を感じなかった　0, 1, 2, 3, 4, 5　強く甘い味を感じた

----------改ページ----------

次に、あなたは口の中で「すっぱい味」がすると言われました。
0から5までの尺度で、どのくらい強くすっぱい味を感じましたか？0は全く感じなかった、5は強くすっぱい味を感じたことを表しています。

あなたの体験に対応する数字を選択してください
全く味を感じなかった　0, 1, 2, 3, 4, 5　強くすっぱい味を感じた

----------改ページ----------

次に、あなたは右腕をまっすぐ伸ばし、硬くなっていくことを感じ、腕を曲げてみるよう指示されました。
0から5までの尺度で、どれほどあなたの腕は硬く感じましたか？0は通常の腕と同じ硬さ、5はどんな力でも曲げられないほど硬く感じたことを表しています。

あなたの体験に対応する数字を選択してください
普通／硬くなかった　0, 1, 2, 3, 4, 5　非常に硬かった

----------改ページ----------

次に、あなたは、あなたの左手と左腕がどれほど重く感じるかを伝えられ、そして手を上げてみるように言われました。
0から5までの尺度で、どのくらいの重さを手に感じましたか？0は全く重さを感じなかった場合、5は非常に重いもので押されているような重さを感じた場合を表しています。

あなたの体験に対応する数字を選択してください
普通／重さを感じなかった　0, 1, 2, 3, 4, 5　非常に重かった

----------改ページ----------

次に、あなたは「ハッピー・バースデー・トゥ・ユー」の曲が聞こえたら、右手を上げてくださいと指示されました。
0から5までの尺度で、どのくらいはっきりと音楽が聞こえましたか？0は曲が全く聞こえなかった場合、5は最高のサウンドシステムから聞こえてくるかのように、はっきりと曲が聞こえた場合を表します。

あなたの体験に対応する数字を選択してください

　音楽は全く聞こえなかった　0, 1, 2, 3, 4, 5　音楽がはっきり聞こえた

----------改ページ----------

次に、あなたは目を開けてカラーボールが 2 つ描かれた絵を見てくださいと言われました。そして、見えたボールの色を入力するように指示されました。
0 から 5 までの尺度で、どの程度 3 つ目のボールが見えにくかったですか？0 は 3 つのボールがはっきりと見えた場合、5 は 2 つのボールしか見えなかった場合を表します。その間の数字は 3 つ目のボールがいくらか見えにくかったことを意味します。

あなたの体験に対応する数字を選択してください
　3 つのボールが見えた　0, 1, 2, 3, 4, 5　2 つのボールが見えた

----------改ページ----------

次に、「あなたは全てを思い出すことができます」と言われるまで、実験中に行ったことは何も思い出せないと言われました。
「あなたは全てを思い出すことができます」と言われるまで、どの程度、この出来事を思い出すことが難しかったですか？0 から 5 までの尺度でお答えください。0 は普段と同じように簡単に思い出すことができたことを意味し、5 はまるで記憶に空白があるかのように、思い出すのが非常に困難だったことを意味します。

あなたの体験に対応する数字を選択してください
　普通に覚えていた　0, 1, 2, 3, 4, 5　全く記憶なし

----------改ページ----------

そのあと、あなたはスペースバーを 6 回連続で押すように指示されましたが、そのことを忘れてしまうと言われました。
スペースキーを押したいという衝動がどの程度あったか、0 から 5 までの尺度でお答えください。0 は衝動が全くないことを意味し、5 はスペースキーを何度も押したいという明確な衝動があったことを表します。

あなたの体験に対応する数字を選択してください。
　衝動はなかった　0, 1, 2, 3, 4, 5　明確な衝動があった

----------改ページ----------

あなたはスペースバーを 6 回連続で押すように言われましたが、そうするように言われたことを忘れてしまうように言われました。
スペースバーを 6 回押すように指示されたことを、どの程度はっきりと覚えているかを 0 から 5 までの尺度で答えてください。0 はその指示を普通に覚えていたこと、5 はその指示を全く覚えていなかったことを意味します。

あなたの体験に対応する数字を選択してください。

指示を普通に覚えていた　0, 1, 2, 3, 4, 5　指示を覚えていなかった

----------改ページ----------

本日の実験の中で、両腕を前に伸ばして手のひらを向かい合わせにするよう指示しました。その際に、実際にそのように体を動かしましたか？

いいえ・はい

----------改ページ----------

実験の中で目を閉じるように指示された際に、実際に目を閉じましたか？

いいえ・はい

----------改ページ----------

本日の実験の中で、「ハッピー・バースデー・トゥ・ユー」の曲が聞こえたら手を挙げてくださいと言われたのを覚えていますか？実際には、このとき一度も曲は流れていませんでした。
また、実験の最後の方で、画面に 2 つのボールが表示されると言いました。このとき、実際には 3 つのボールが画面には見えていました。
この 2 つの項目の目的は、あなたをだますことではありません。これまでの研究によって、主観的な経験を制御することに長けている人は、暗示によって自分の知覚（見ているものや、聞いているもの）を変えることができるとわかっています。この 2 つの項目は、あなたが知覚を変えてしまえる能力を評価するためのものです。
