# Supplementary material for: The Japanese version of the Phenomenological Control Scale
Source: Neurosci Conscious. 2025 May 21;2025(1):niaf008. doi: 10.1093/nc/niaf008 (PMC12094076; doi:10.1093/nc/niaf008)
Supplement: niaf008_Supp [file niaf008_supp.zip › suppl_data/PCS-J_Script_Text_v2.pdf]

The Japanese version of the Phenomenological Control Scale
日本語版現象学的制御尺度

読み上げ教示文

備考

- 次頁以降における丸括弧内の言葉は読み上げない。

参考文献

Lush, P., & Dienes, Z. (2022, April 20). Phenomenological Control Scale norms. Retrieved from https://osf.io/4x25a/

Lush, P., Scott, R. B., Seth, A. K., & Dienes, Z. (2021). The Phenomenological Control Scale: Measuring the capacity for creating illusory nonvolition, hallucination and delusion. *Collabra: Psychology, 7*(1), 29542. https://doi.org/10.1525/collabra.29542

（1.導入.wav）

これから、想像力を使うことで、様々な体験を作り出すための練習をします。
この練習の目的は、「手が動いている」というような単純なできごとの体験のしかたを、どれだけ自分でコントロールできるかを調べることにあります。
例えば、あなたに手を下げるようにお願いしたら、その手がひとりでに下に引っ張られていくのを想像してください。
手がひとりでに下に引っ張られるような感じがするまで、その想像に集中してください。
手の感覚と想像している場面に集中することで、映画の物語に夢中になるように、まるで現実であるかのように、その体験に没頭することができます。
そのあと、それぞれの体験の強さ、例えば、どれほど手が勝手に動いたように感じたかを評価してもらいます。
この練習が上手くいくかは、私が想像するようにお願いしたことを、あなたが進んで想像し、はっきりと心にイメージを浮かべようとするかどうかにかかっています。
私が説明することを、できるだけ生き生きと想像して、この実験に協力してください。

では、楽な姿勢で座ってください。
両手を太ももの上に置いてください。

そうです。
では、目を閉じて、私の声だけに集中してください。
簡単な想像力の練習でウォーミングアップします。
今から 1 から 20 まで数字を数えます。
私が数を数えるごとに、あなたは階段を下りていくところを想像してください。
一歩一歩、足を踏み出すのを見て、それを感じてください。
一、あなたは階段を一段下ります。
二、もう一歩。
三。
四。
階段を一段、一段下りていきます。
五。
六。
七。
あなたは想像に没入できることに気づくと思います。
何も邪魔するものはありません。
ただ、あなたの思考を、私の声と私が言っていることに集中するようにしましょう。
私の言うことを楽に聞けるように感じてきます。

八。
九。
十。
いま半分まできました。
十一。
十二。
十三。
十四。
十五。
想像に没入していると、私の声がはっきり聞こえます。
十六。
十七。
十八。
階段を下りる一歩一歩によく集中しましょう。
あなたは私が言うとおりの体験をするようになります。
十九。
二十。
はい、いいでしょう。
では練習を始めましょう。
この練習が終わったあと、体験したことを、コンピューター上で報告していただきます。
それでは始めましょう。

（2.暗示.wav）

（手が下がる）

（数秒待つ）
右手を肩の高さまであげて、手のひらを上に向けてください。
右手を体からまっすぐに伸ばして、手のひらは上に向けてください。
そうです。
右手に意識を集中してください。
右手の感覚や、手に何が生じたかを注意深く感じてください。
わずかにしびれている感じや、ヒリヒリした感じ、
手首を曲げないようにするためのわずかな努力、
手にあたる風を感じるかどうか、注意してみてください。
今、この瞬間の、あなたの手に意識を集中してください。
何か重いものを手に持っていると想像してください。
ボーリングの球のような、何か重いものです。

この重いものを手に持っているかのように、右手をお椀のような形にしてみましょう。

そうです。いいですね。
あなたは、手と腕に重さを感じてきました。
まるで重りが手を押しているようです。
その重りは、まるでどんどん重くなっているかのようです。
それに従って、手が下に下がっていきます。
まるで、重りが手を下に押し付けているかのように、手が下がっていきます。
腕が下がっていきます。
もっと、もっと下に、手が下がっていきます。
さらに重く、おもーくなっていきます。
腕はさらに疲れてきました。
腕が下がっていきます。
ゆっくりと、でも確実に下がっていきます。
下に、下に、もっと下がっていきます。
この重りは、とっても重いです。
手が本当に重くなっていきます。
あなたは重さをさらに感じるようになります。
もっと重くなります。
腕が重くなりすぎて、しっかりと支えることができません。
さらに下へ下へと下がっていきます。
腕がもっと下がっていきます。ずうっと下がっていきます。
（10 秒待つ）
いいでしょう。
それでは、手を元の位置に戻してみましょう。
あなたは、おそらく腕に重さと疲れを感じたでしょう。
その感じられた重さや疲れは、腕を下に下げようとするものを想像しなかったり、腕に集中していなかった場合よりも大きなものだったでしょう。
あなたの手と腕は、今、疲労感も緊張感もなく、元通りになっています。

（手が互いにくっつく）

（数秒待つ）
今度は両腕を前に伸ばして、手のひらを向かい合わせにします。
両手を 30 センチほど離して、お互いに向けます。
今から、あなたの両手を引き寄せるような力が生じていると思ってみてください。
片方の手が、もう片方の手を引き寄せているかのようにです。

あなたは、両手が引っ張られて、動き始めるところを想像します。
両手はお互いに寄っていきます。
両手が近づいていきます。
お互いに吸い寄せられていきます。
もっと近づいていきます。
もっともっと近づいていきます。
さらに寄っていきます。
（10 秒待つ）
いいでしょう。
あなたの想像と、実際の動作が、いかに密接に繋がっているかに気づいたことでしょう。
では、両手を元の位置に戻してください。

（蚊の幻覚）

（数秒待つ）
ここまで、私の話を注意深く聞いてくれていますね。
あなたは、さっきからずっと、蚊がブーンと飛び回って、音をたてていたことに気づいていないかもしれません。
その蚊の音を聞いてください。
その甲高いブーンという音を聞いてください。
あなたの右手の周りを飛びまわっています。
蚊は、あなたの手の上に降り立ちました。
手が少しくすぐったいです。
そして、飛び立ちます。
また手にとまりました。
刺されるかもしれません。
この蚊がうっとうしいです。
あなたは、追い払いたいと思っています。
さぁ、追い払ってください。
気になるなら追い払ってください。
（10 秒待つ）
蚊はいなくなりました。
もう気になりません。
蚊は消えました。

（味の幻覚）

（数秒待つ）

口の中に甘いものを思い浮かべてください。
甘い味のするもの、例えば小さな砂糖のかたまりが口の中にあると想像してください。
この甘い味を思い浮かべているうちに、実際に甘い味がしてきます。
最初は微かかもしれませんが、だんだんと味が強くなっていきます。
甘く、あまーくなっていきます。
今、あなたは口の中に甘い味を感じ始めました。
口の中がどんどん甘くなっていきます。
甘く、あまーく。
どんどん甘くなっていきます。
はっきりと甘みを感じるまで、少しかかるかもしれませんが、甘みはどんどん強くなっていきます。
甘く、あまーくなっていきます。
（10 秒待つ）
さて、いいでしょう。
先ほどまでの味がなにか変わったか気づきましたね。
味が変わっています。
口の中に酸っぱい味がしてきましたね。
酸っぱい味、まるでレモンを口に含んだか、お酢を口にいれたかのような味です。
口の中が、どんどん酸っぱくなってきました。
より酸っぱくなります。
さっきよりも酸っぱくなりました。
もっともっと酸っぱくなっていきます。
（10 秒待つ）
はい。
酸っぱい味は消えました。
口の中は、私が味について話す前と同じように感じるはずです。
あなたの口の中は元通りになりました。
ごく普通に戻りました。

（腕の硬さ）

（数秒待つ）
右腕をまっすぐ前に出してください。
指もまっすぐ伸ばしてください。
そうです。
右腕をまっすぐ伸ばしてから、あなたの腕がどんどん硬くなっていくのを想像してください。

硬く、かたぁく、非常に硬くなっていきます。
硬くなっていくのを想像すると、実際に腕が硬くなるのを感じるでしょう。
もっと硬く、かたぁく。
まるで肘が曲がらないように添え木をしたようです。
硬く、硬くなっていきます。
きつく縛られた腕は、曲がりません。
添え木できつく縛られた腕は全く曲がりません。
あなたの腕は、きつく添え木をしているように硬く感じられます。
どのくらい硬いか、試してみましょう。
腕を曲げようとしてみてください。
さぁ試してみてください。
（10 秒待つ）
それで結構です。
この後も、いろいろなことを経験していきます。
おそらくあなたは、腕が硬くなると想像しているうちに、どれほど腕が硬くなったかに気づきましたね。
また、その腕を曲げるのにどれほどの努力が必要だったかにも気づきましたね。
あなたの腕はもう全然硬くありません。
元の位置に戻してください。

（腕の不動）

（数秒待つ）
さて、あなたの左手は膝の上にあるはずです。
ここでは、あなたの左腕と左手について考えてみてください。
左腕と左手に意識を集中してください。
しびれて、重い感じがします。
とても重いです。
左手がなんて重いのでしょう。
左手がどれだけ重いか考えている間にも、左手はどんどん、どんどん重くなっていきます。
あなたの手はさらに重くなってきました。
もっと重く、おもーくなります。
とても、とても重くなってきます。
あなたの手はさらに重くなります。
非常に重いです。
まるで膝に押し付けられているようです。
あなたの手がどれだけ重いか、ちょっと確かめてみたいと思うかもしれません。

重すぎて動かせないようです。
しかし、これだけ重いのに、もしかしたら少しは動かせるかもしれません。
でも、それすらもできないくらい重いかもしれません。
どれだけ重いか試してみましょう。
手を上に上げてみてください。
どうぞ試してみてください。
（数秒待つ）
はい、いいでしょう。
では、手を元の位置に戻してください.
あなたの手と腕は、今は普通に感じられます。
もはやまったく重くありません。

（音楽の幻覚）

（数秒待つ）
まもなく、「ハッピー・バースデイ・トゥー・ユー」の曲が再生されます。
音声が流れ始めるときには音量はかなり小さく、おそらく、あなたには聞こえないでしょう。
聞こえたとしても、非常にかすかにしか聞こえないはずです。
その後、音量が大きくなっていくので、曲が聞こえるようになったら、右手を上げて知らせてください。
いいでしょうか？
ではいきましょう。
「ハッピー・バースデイ・トゥー・ユー」の再生を始めました。
これがレベル 1 です。
（5 秒待つ）
今、少し音量を上げました。
これがレベル 2 です。
聞こえる人は手を上げてください。
（5 秒待つ）
さらに音量を大きくしていきます。
これはレベル 3 です。
（5 秒待つ）
では、今から一番大きな音量にします。
これはレベル 4 です。
音楽が聞こえたら手を上げてください。
（5 秒待つ）

さて、音楽は止まりました。
今はもう曲は流れていません。
手を元の位置に戻してください。
では、座ったままでいてください。

（3.幻視はじめに.wav）

（数秒待つ）
あなたは、目を閉じて、椅子にゆったりと座っています。
これから、コンピューターの画面に、2 つのボールの絵が表示されます。
2 つの色の付いたボールは、あなたの目の前の画面にはっきりと表示されるので、よく見えると思います。
この後少ししたら、目を開けるように言います。
画面には 2 つのボールだけが表示されます。
2 つのボールだけです。

（4.幻視ボール.wav）

（カラーボール画像 https://osf.io/sc4d2 を提示）
さぁ、目を開けて、画面を見てください。
画面に見える 2 つのボールをよく見ましょう。
今あなたが見ているボールの色を記憶しておいてください。
後でボールの色を報告してもらうので、見えたボールの色を覚えておいてください。
では、目をつぶってください。

（5.幻視目を開ける.wav）

（数秒待つ）
それでは、目を開けて、あなたが見たボールの色をコンピューターに入力してください。
見えたボールの色を書いてください。

（6.幻視入力.wav）

ボールの色を入力し終わったら、「完了」を押してください。
終わったら、腕を元の位置に戻してください。
（入力完了を待つ）

（7.1.記憶喪失 1.wav）

あなたは、ここまでとてもよくやっています。
目を閉じたままにしてください。

次の練習にいきましょう。
あなたが最初に目を閉じてから、今まで私があなたに話したこと、あなたがしたこと、感じたことを思い出せなくなります。
実際、これらのことを思い出すのはとても努力のいることで、思い出したいとは思わないでしょう。
私が「思い出せる」と言うまで、何もかも忘れてしまう方がずっと楽です。
目を閉じてから、私が「さあ、あなたは全てを思い出すことができます！」と言うまで、あなたは自分が何をしたのか、何を感じたのか、何も覚えていないでしょう。
「思い出せる」と聞くまで、あなたは自分のやったことを忘れてしまいます。
私が「目を開けてください」と言うと、しばらくして、画面に「お待ちください」というメッセージが表示されます。
画面に「お待ちください」と表示されたら、スペースバーを 6 回押してください。
あなたはスペースバーを 6 回押しますが、私がそうするように言ったことを忘れてしまいます。
他のことを忘れるのと同じように、私が「あなたは全てを思い出すことができます！」と言うまで、そのことを思い出せません。
さあ、目を開けてください。
（画面に「（お待ち下さい）」と 10 秒間表示する）
（画面に「この実験を始めてから今までに起こったことを簡単に書き出してください。細部まで書く必要はありません。２分間で書き終えてください。時間がきたら自動的に次に進みます。」と 2 分間まで表示する）

（8.思い出せる.wav）

今から言う私の言葉を注意深く聞いてください。
さあ、あなたは全てを思い出すことができます。
以前は思い出せなかったことで、今思い出したことがあれば、それを書いてください。
詳細を書く必要はありません。
書く時間は 2 分間で、それ以上時間をかけないでください。
先ほどと同じように、時間になったらお知らせします。
