# Supplementary material for: The Japanese version of the Phenomenological Control Scale
Source: Neurosci Conscious. 2025 May 21;2025(1):niaf008. doi: 10.1093/nc/niaf008 (PMC12094076; doi:10.1093/nc/niaf008)
Supplement: niaf008_Supp [file niaf008_supp.zip › suppl_data/PCS-J_Suppl_Results.pdf]

Supplementary results for "The Japanese version of the Phenomenological Control Scale"

Shu Imaizumi, Keisuke Suzuki

Below are robustness checks of the Bayes factor (BF_{10}) for Pearson's correlation and Student's *t*-test performed by JASP 0.19.2 (JASP Team, 2024). All analyses are two-tailed.

Test-retest reliability

Figure S1. Robustness check for paired *t*-test comparing PCS-J scores in Surveys 1 and 2.

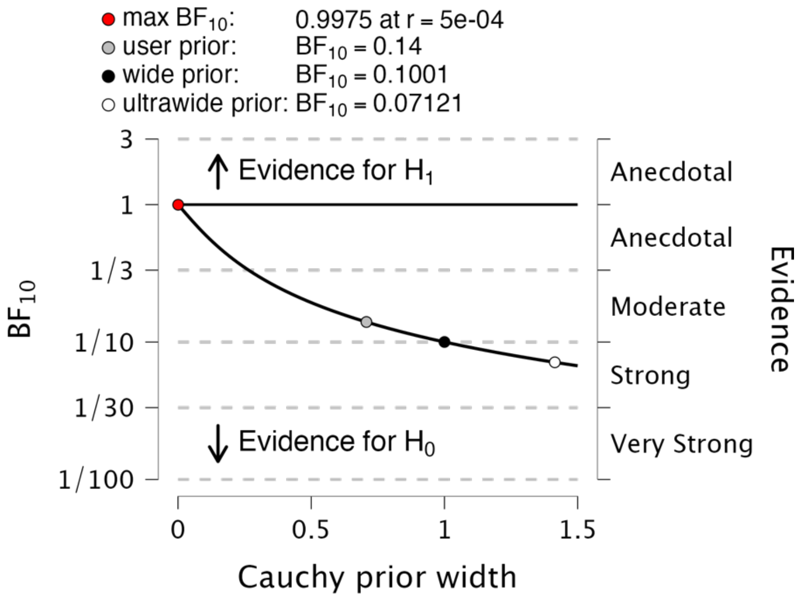

Figure S2. Robustness check for correlation between PCS-J scores in Surveys 1 and 2.

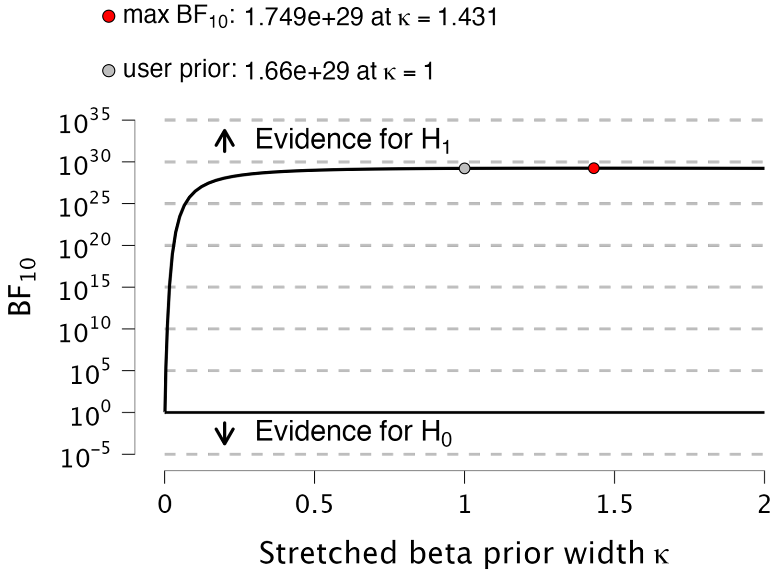

Construct validity

Figure S3. Robustness check for correlation between PCS-J and SPQ-B Total scores.

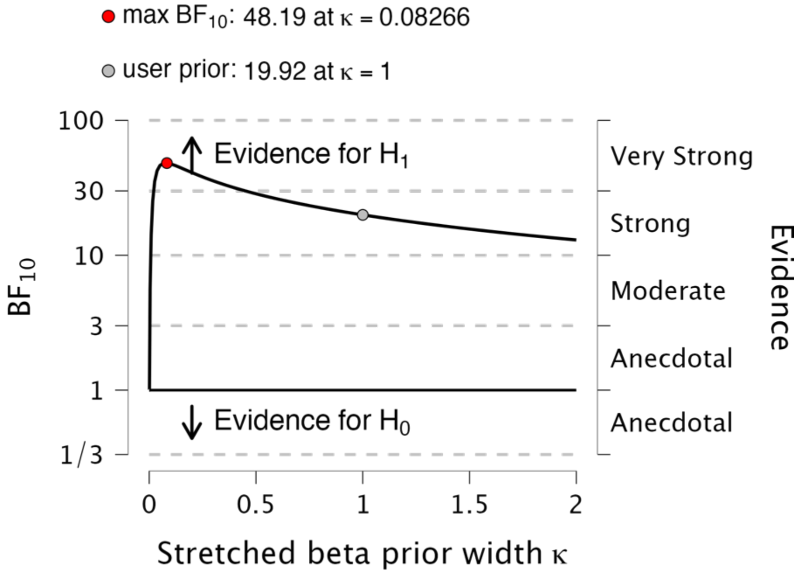

Figure S4. Robustness check for correlation between PCS-J and SPQ-B Cognitive-Perceptual scores.

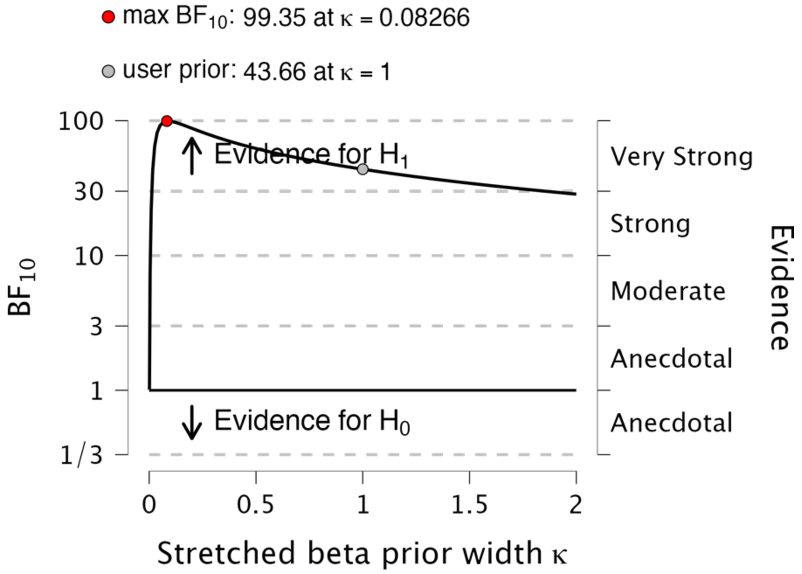

Figure S5. Robustness check for correlation between PCS-J and SPQ-B Interpersonal scores.

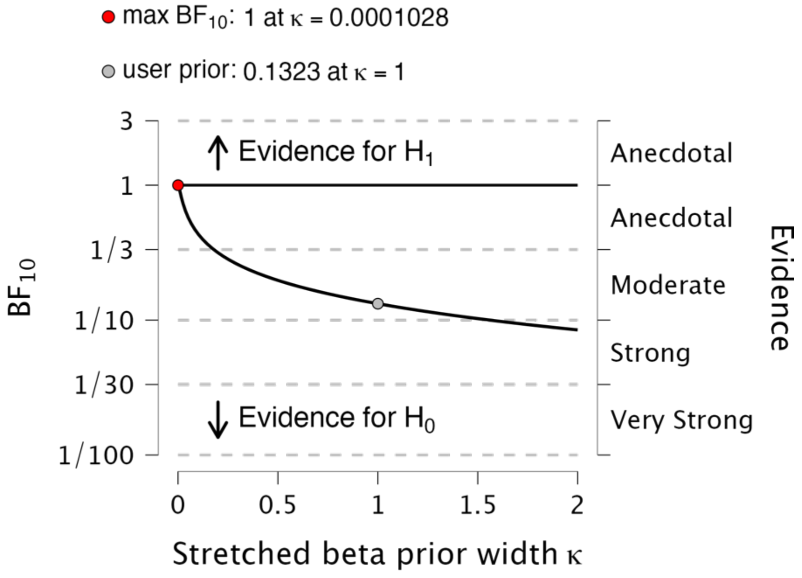

Figure S6. Robustness check for correlation between PCS-J and SPQ-B Disorganized scores.

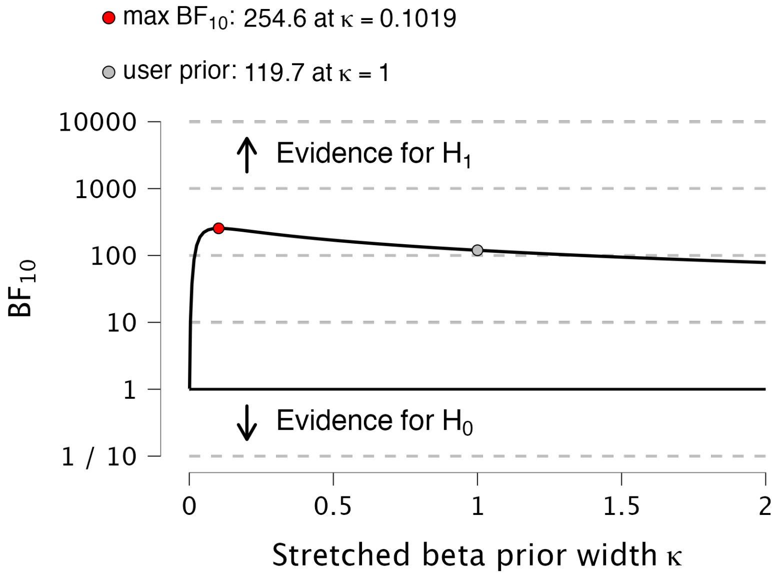

Comparison with English versions

Figure S7. Robustness check for unpaired *t*-test comparing PCS-J and SWASH (Palfi et al., 2020) scores.

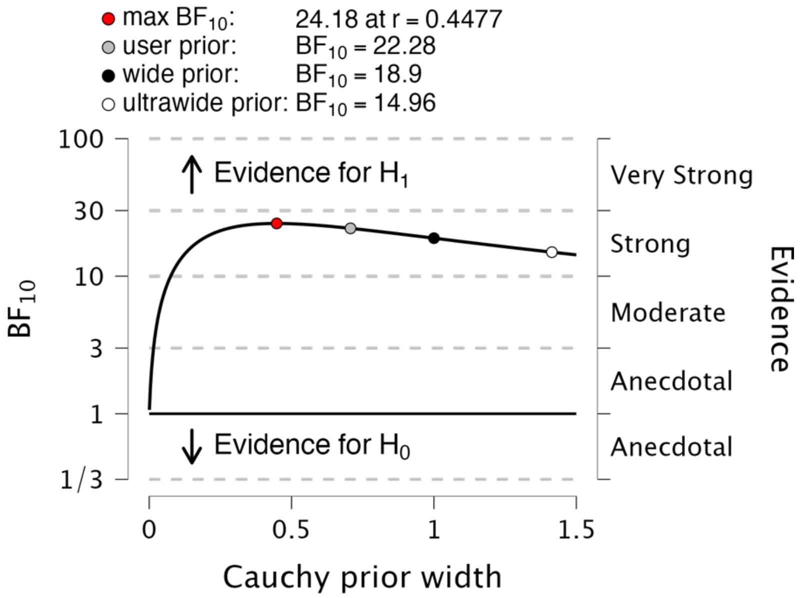

Figure S8. Robustness check for unpaired *t*-test comparing PCS (Lush et al., 2021) and SWASH (Palfi et al., 2020) scores.

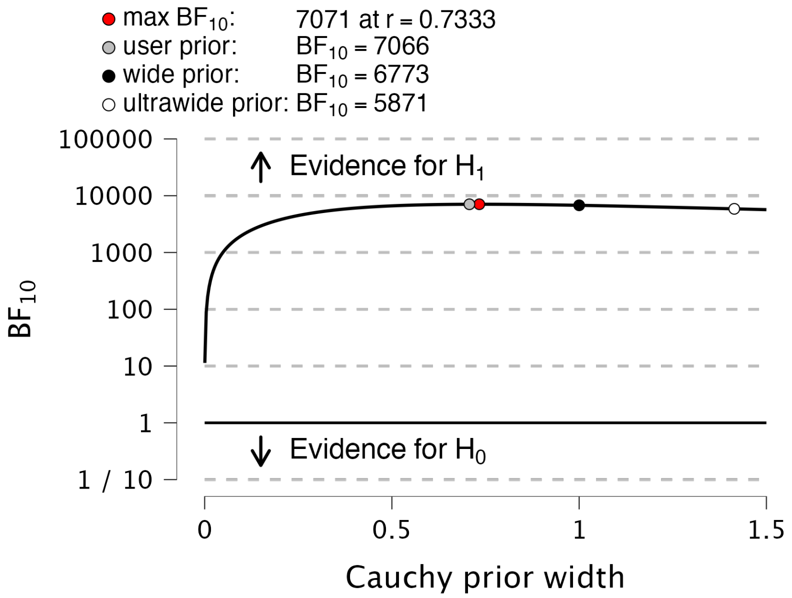

Figure S9. Robustness check for unpaired *t*-test comparing PCS-J and PCS (Lush et al., 2021) scores.

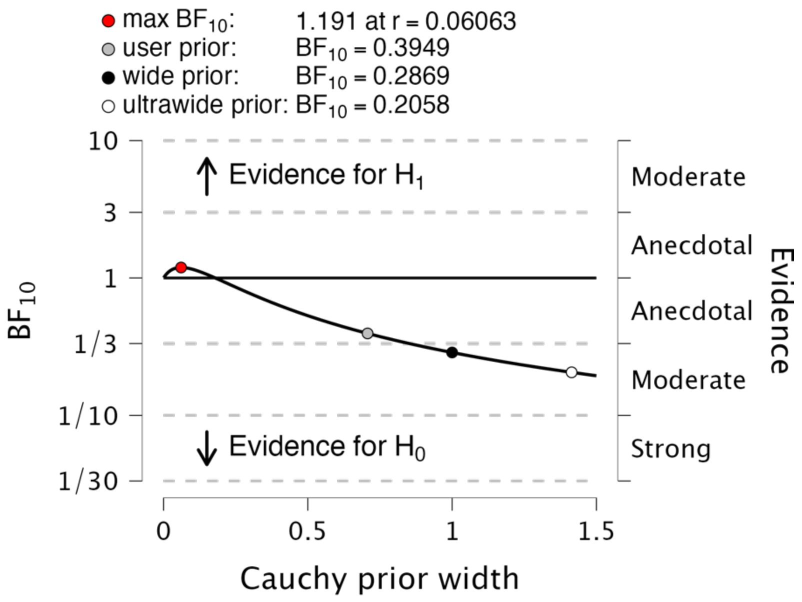

Abbreviations

PCS: Phenomenological Control Scale (Lush et al., 2021)

PCS-J: Japanese version of the Phenomenological Control Scale

SWASH: Sussex-Waterloo Scale of Hypnotizability (Lush et al., 2018)

SPQ-B: Schizotypal Personality Questionnaire Brief (Ito et al., 2008; Raine & Benishay, 1995)

References

Ito, S., Obu, S., Ota, M., Takao, T., & Sakamoto, S. (2008). Reliability and validity of the Japanese version of SPQ-B (Schizotypal Personality Questionnaire Brief). *Japanese Bulletin of Social Psychiatry, 17*(2), 168-176.

JASP Team. (2024). *JASP version 0.19.2*. Retrieved December 10, 2024 from https://jasp-stats.org

Lush, P., Moga, G., McLatchie, N., & Dienes, Z. (2018). The Sussex-Waterloo Scale of Hypnotizability (SWASH): Measuring capacity for altering conscious experience. *Neuroscience of Consciousness, 2018*(1), niy006. https://doi.org/10.1093/nc/niy006

Lush, P., Scott, R. B., Seth, A. K., & Dienes, Z. (2021). The Phenomenological Control Scale: Measuring the capacity for creating illusory nonvolition, hallucination and delusion. *Collabra: Psychology, 7*(1), 29542. https://doi.org/10.1525/collabra.29542

Palfi, B., Moga, G., Lush, P., Scott, R. B., & Dienes, Z. (2020). Can hypnotic suggestibility be measured online? *Psychological Research, 84*(5), 1460-1471. https://doi.org/10.1007/s00426-019-01162-w

Raine, A., & Benishay, D. (1995). The SPQ-B: A brief screening instrument for schizotypal personality disorder. *Journal of Personality Disorders, 9*(4), 346-355. https://doi.org/10.1521/pedi.1995.9.4.346
